# A human polymorphism affects NEDD4L subcellular targeting by leading to two isoforms that contain or lack a C2 domain

**DOI:** 10.1186/1471-2121-10-26

**Published:** 2009-04-13

**Authors:** Nicholas F Garrone, Bonnie L Blazer-Yost, Robert B Weiss, Jean-Marc Lalouel, Andreas Rohrwasser

**Affiliations:** 1 Department of Human Genetics, Eccles Institute of Human Genetics, University of Utah School of Medicine, Salt Lake City, USA; 2 Department of Biology, Indiana University Purdue University at Indianapolis, Indianapolis, USA

## Abstract

**Background:**

Ubiquitination serves multiple cellular functions, including proteasomal degradation and the control of stability, function, and intracellular localization of a wide variety of proteins. NEDD4L is a member of the HECT class of E3 ubiquitin ligases. A defining feature of NEDD4L protein isoforms is the presence or absence of an amino-terminal C2 domain, a class of subcellular, calcium-dependent targeting domains. We previously identified a common variant in human *NEDD4L *that generates isoforms that contain or lack a C2 domain.

**Results:**

To address the potential functional significance of the *NEDD4L *common variant on NEDD4L subcellular localization, NEDD4L isoforms that either contained or lacked a C2 domain were tagged with enhanced green fluorescent protein, transfected into *Xenopus laevis *kidney epithelial cells, and imaged by performing confocal microscopy on live cells. We report that the presence or absence of this C2 domain exerts differential effects on the subcellular distribution of NEDD4L, the ability of C2 containing and lacking NEDD4L isoforms to mobilize in response to a calcium stimulus, and the intracellular transport of subunits of the NEDD4L substrate, ENaC. Furthermore, the ability of the C2-containing isoform to influence β-ENaC mobilization from intracellular pools involves the NEDD4L active site for ubiquitination. We propose a model to account for the potential impact of this common genetic variant on protein function at the cellular level.

**Conclusion:**

NEDD4L isoforms that contain or lack a C2 domain target different intracellular locations. Additionally, whereas the C2-containing NEDD4L isoform is capable of shuttling between the plasma membrane and intracellular compartments in response to calcium stimulus the C2-lacking isoform can not. The C2-containing isoform differentially affects the mobilization of ENaC subunits from intracellular pools and this trafficking step requires NEDD4L ubiquitin ligase activity. This observation suggests a new mechanism for the requirement for the PY motif in cAMP-mediated exocytosis of ENaC. We have elucidated how a common genetic variant can underlie significant functional diversity in NEDD4L at the cellular level. We propose a model that describes how that functional variation may influence blood pressure. Moreover, our observations regarding differential function of the NEDD4L isoforms may impact other aspects of physiology that involve this ubiquitin ligase.

## Background

Cells of various tissues employ ubiquitination to regulate multiple proteins including cell cycle regulators, transcription factors, and membrane proteins [1,2]. Despite the diverse nature of ubiquitinated proteins, ubiquitination occurs in a common three step process [3]. First an ubiquitin activating, or E1 enzyme, binds ubiquitin in an ATP-dependent-process. Second, ubiquitin is transferred to an ubiquitin conjugating, or E2 enzyme. Third, an ubiquitin ligase or E3 enzyme catalyzes the transfer of ubiquitin to its substrate. There are three classes of E3 ubiquitin ligases, HECT (homologous to E6-AP carboxy terminus) E3 ligases, RING-finger E3 ligases [4,5], and U-box E3 ligases [6,7]. Whereas HECT E3 ligases directly bind ubiquitin prior to catalyzing substrate ligation, RING-finger and U-box E3 ligases do not bind ubiquitin, they function as adaptors, providing a physical link between the E2 enzyme and the substrate, catalyzing the transfer of ubiquitin directly from the E2 enzyme to the substrate [8].

**Figure 1:**
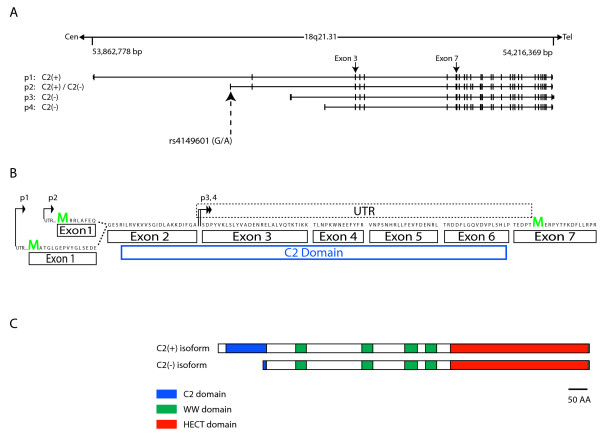
**NEDD4L is expressed as isoforms that contain (NEDD4L-C2(+)) or lack (NEDD4L-C2(-)) a calcium binding C2 domain**. (A) Chromosome 18q21.31 where *NEDD4L *transcription occurs from several major alternate promoters designated p1-4 and generates NEDD4L-C2(+) and NEDD4L-C2(-) isoforms. Several additional promoters that generate NEDD4L-C2(+) and NEDD4L-C2(-) isoforms exist [11]. The location of rs4149601 is labeled on the p2 transcript. (B) The C2(+) domain transcript that arises from p1 encodes a methionine in exon 1, initiating translation upstream of the C2 domain thereby generating NEDD4L-C2(+) isoforms. The p2 transcript generates NEDD4L-C2(+) and NEDD4L-C2(-) isoforms depending on the identity of the rs4149601 variant [11]. Whereas the G variant generates NEDD4L-C2(+) and NEDD4L-C2(-), the A variant only generates NEDD4L-C2(-) [11]. Transcripts from p3 and p4 encode only NEDD4L-C2(-) utilizing an initiation codon in exon 7, downstream of the C2 domain. (C) The domain architecture of human NEDD4L-C2(+) and NEDD4L-C2(-). Each isoform contains four WW domains and a HECT (homologous to E6 associated protein carboxy terminus) domain.

NEDD4L (neural precursor cell-expressed developmentally down-regulated 4like) is a member of the Nedd4-like family of E3 ubiquitin ligases. The nine members of the Nedd4-like family in humans share a common domain organization that is evolutionarily conserved in the *S. cerevisiae *E3 ligase ortholog, Rsp5 [9,10]. Each contains two to four WW domains, which mediate substrate interactions, and a carboxy terminal HECT domain, that catalyzes ubiquitin ligation and mediates interactions with E2 enzymes [10]. Additionally, each family member contains a C2 domain–functionally known for its calcium dependent lipid binding ability–at its amino terminus. The discovery of multiple promoters that generate transcripts that contain a start codon either upstream or downstream of the C2 domain, reveals that in humans and rodents, NEDD4L can be expressed as a protein containing or lacking a C2 domain (Figure 1A–C) [11-13]. The evolutionary conservation of C2 containing or lacking NEDD4L isoforms across multiple vertebrates could underlie functional variability that could differentially affect NEDD4L substrates.

The epithelial sodium channel (ENaC) is a heteromultimeric transmembrane ion channel consisting of alpha, beta and gamma (α, β and γ) subunits that is a critical mediator of sodium absorption in the epithelium of multiple tissues. ENaC expression at the apical side of the epithelium lining the lumen of the aldosterone-sensitive distal nephron (ASDN) positions ENaC as the ultimate determinant of renal sodium excretion [14]. It is thereby a critical factor in blood pressure regulation [14]. Indeed, loss- or gain-of-function in ENaC subunits accounts for rare Mendelian forms of hypo- or hypertension [15-23]. Pseudohypoaldosteronism type I (PHAI) is an autosomal recessive form of hypotension that is caused by loss-of-function mutations in either the α-, β- or γ-ENaC subunit [15,16,21]. Liddle syndrome, in contrast, is an autosomal dominant form of hypertension that is caused by gain-of-function mutations that alter or delete PY motifs in the cytoplasmic tails of either the β- or γ-ENaC subunit [17-20,22,23]. These PY motifs interact with WW domains of NEDD4L [24,25], and the PY motif mutations cause gain-of-function by increasing the cell-surface expression of ENaC. Liddle's mutations are consistent with increased ENaC activity at [26], an increase in the number of channels in [27], and reduced ENaC endocytosis from the membrane [28]. Liddle's PY motif mutations also inhibit cAMP-mediated rapid translocation of ENaC from intracellular pools to the cell surface stimulated by vasopressin binding to the V2 vasopressin receptors [29]. Combining these observations with those that demonstrate that ENaC is ubiquitinated by NEDD4L [30] and that NEDD4L and ENaC expression in the ASDN overlap [30,31] suggested a mechanism that underlies Liddle syndrome. Impaired interaction between ENaC and NEDD4L due to mutated PY motifs reduces NEDD4L-mediated ENaC ubiquitination, thereby decreasing ENaC endocytosis from the plasma membrane, leading to abnormally high sodium retention and ultimately hypertension.

Essential hypertension, a major contributor to cardiovascular morbidity and mortality [32], involves multiple genetic and environmental determinants [33]. This complex etiology prompted genetic analyses of intermediate blood pressure phenotypes [34]. A significant linkage peak for postural changes in blood pressure on chromosome 18q from a study conducted by the Hypertension Genetic Epidemiology Network contains human *NEDD4L *[35,36]. We previously identified a common single nucleotide polymorphism (SNP, rs4149601, G/A, hereafter referred to as the "G/A variant") at the last nucleotide of exon 1 in *NEDD4L *that alters exon 1 splice donor site selection [11] (Figure 1A–B). Whereas the G variant leads to splicing of distinct mRNAs encoding protein isoforms that either contain or lack the C2 domain (NEDD4L-C2(+) and NEDD4L-C2(-) respectively), the A variant only produces mRNAs that encode NEDD4L-C2(-) (Figure 1A–B). Subsequent association studies implicate the G/A variant in blood pressure variation and salt sensitivity [37-40].

The implication of NEDD4L in Liddle syndrome, genetic linkage of *NEDD4L *to a blood pressure phenotype, and evidence that ties the G/A variant to blood pressure variation and salt sensitivity, supported the hypotheses that NEDD4L participates in blood pressure regulation and that genetic variation in *NEDD4L *such as the G/A variant could contribute to individual differences to susceptibility to essential hypertension. We investigated the functional relevance of the G/A variant by examining the localization behavior of NEDD4L-C2(+) and NEDD4L-C2(-) in *X. laevis *kidney epithelial (A6) cells in the absence and presence of a calcium stimulus. Published evidence regarding the functional interactions between NEDD4L and ENaC [41-44] and the availability of two clonal A6 cell lines that stably expressed functional α- or β-ENaC subunit as EGFP fusion proteins [45], provided the rationale and a cellular model to test the effect(s) of potential functional differences between NEDD4L-C2(+) and Neddd4l-C2(-) on the NEDD4L substrates α- or β-ENaC. We report that NEDD4L-C2(+) and Neddd4l-C2(-) isoforms exhibit distinct subcellular distributions and exert differential affects on the intracellular transport of ENaC subunits that depend on the ubiquitin ligase activity of the HECT domain. We propose a model to account for the potential functional impact of the G/A variant on sodium handling and blood pressure regulation involving NEDD4L and ENaC.

## Results

### NEDD4L-C2(+) localizes to the cytoplasm while NEDD4L-C2(-) localizes to the cytoplasm and early endosome

**Figure 2:**
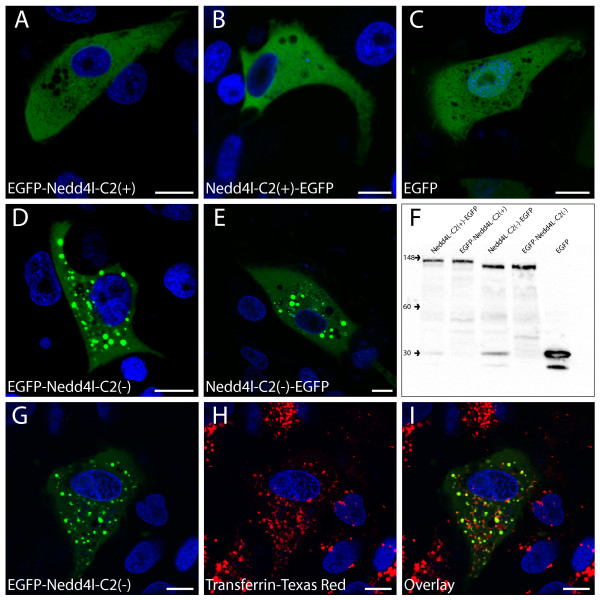
**Differential subcellular localization of EGFP tagged NEDD4L-C2(+) and NEDD4L-C2(-) isoforms**. Confocal microscopic images of *X. laevis *A6 cells transiently transfected with EGFP-NEDD4L-C2(+) (A), NEDD4L-C2(+)-EGFP (B), EGFP (C), EGFP-NEDD4L-C2(-) (D), and NEDD4L-C2(-)-EGFP (E). Western blot experiments of crude lysates from transiently transfected A6 cells (F) demonstrate that differential degradation of either isoform did not occur. The monoclonal EGFP antibody, JL-8 (Clontech) was used for detection. Confocal microscopic images of A6 cells transiently transfected with EGFP-NEDD4L-C2(-) and incubated with the early endosomal marker, Transferrin-Texas Red^® ^(G-I) indicate that EGFP-NEDD4L-C2(-) localizes to the early endosome. Green (EGFP-tagged NEDD4L-C2(-)) and blue (nuclear marker) channels (G). Red (early endosome marker) and blue channels (H). Green, blue and red channel overlay (I). Scale bars are equivalent to 10 μm.

To address the potential functional significance of the G/A variant on NEDD4L subcellular localization, NEDD4L-C2(+) and NEDD4L-C2(-) were tagged with enhanced green fluorescent protein (EGFP), transfected into A6 (*X. laevis *kidney epithelial) cells, and imaged by using live cell confocal microscopy. EGFP-NEDD4L-C2(+) exhibited a diffuse cytoplasmic localization, similar to EGFP alone (Figure 2A–C). In addition to this cytoplasmic localization, EGFP-NEDD4L-C2(-) exhibited a distinct compartmental localization (Figure 2D–E). These phenotypes were independent of the N- or C-terminal fusion of the EGFP reporter (Figure 2A–B, D–E) and did not reflect differential degradation of the two isoforms in Western blot experiments (Figure 2F). The EGFP-NEDD4L-C2(-) compartments partially colocalized with the early endosome marker transferrin-Texas Red^® ^(Figure 2G–I) but not with other organelle markers tested (see Additional file 1). We therefore conclude that when transiently expressed, EGFP-NEDD4L-C2(+) localizes to the cytoplasm while EGFP-NEDD4L-C2(-) localizes to the cytoplasm and to early endosomal compartments in A6 cells.

### The presence or absence of a C2 domain affects intracellular NEDD4L targeting in response to an increase in intracellular calcium

**Figure 3:**
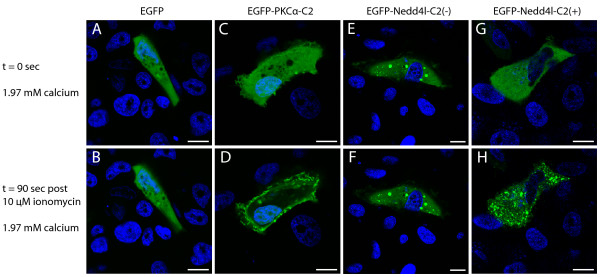
**Subcellular localization of EGFP tagged NEDD4L-C2(+) and NEDD4L-C2(-) isoforms in response to ionomycin and Ca^2+ ^treatment**. Confocal microscopic images of live A6 cells transiently transfected with EGFP (A, B), EGFP-PKCα-C2 (C, D), EGFP-NEDD4L-C2(-) (E, F), and EGFP-NEDD4L-C2(+) (G, H). Images prior to (A, C, E & G) and 90 seconds after (B, D, F, H) incubation with10 μM ionomycin and 1.97 mM Ca^2+^. EGFP and EGFP-NEDD4L-C2(-) do not relocalize in response to an intracellular Ca^2+ ^influx. EGFP-PKCα-C2 relocalizes to the plasma membrane. EGFP-NEDD4L-C2(+) mobilizes to numerous small intracellular structures. Scale bars are equivalent to 10 μm.

**Figure 4:**
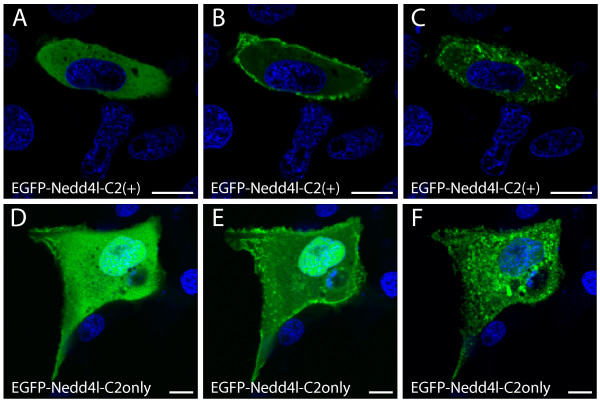
**Subcellular localization of the EGFP tagged NEDD4L-C2(+) isoform and the NEDD4L C2 domain (C2(only)) in response to ionomycin and Ca^2+ ^treatment**. Confocal images of live A6 cells transiently transfected with EGFP-NEDD4L-C2(+) (A-C) or EGFP-C2(only) (D-F) that were incubated in 1.97 Ca^2+^. Images were acquired at 0 (A), 40 (B), 90 (C), 0 (D), 10 (E), and 30 (F) seconds after a 10 uM ionomycin addition. Both EGFP-NEDD4L-C2(+) and EGFP-C2(only) respond to the Ca^2+ ^stimulus by first mobilizing to the plasma membrane and then relocalizing to numerous small intracellular structures. Scale bars are equivalent to 10 μm.

To test the calcium-dependent lipid targeting capability of the NEDD4L C2 domain, A6 cells were transfected with EGFP-NEDD4L-C2(+) and EGFP-NEDD4L-C2(-), incubated in the presence of Ca^2+ ^and ionomycin, and imaged. The protein kinase C alpha (PKCα) C2 domain fused to EGFP served as positive control (EGFP-PKCα-C2) for calcium-dependent plasma membrane targeting [46]. Prior to ionomycin treatment, EGFP-PKCα-C2, EGFP-NEDD4L-C2(+) and the EGFP control localized to the cytoplasm while EGFP-NEDD4L-C2(-) localized to the cytoplasm and early endosome (Figure 3A, C, E, G). Upon incubation with Ca^2+ ^and ionomycin, EGFP remained in the cytoplasm while EGFP-PKCα-C2 moved to the plasma membrane (Figure 3B, D). EGFP-NEDD4L-C2(-) was unaffected by Ca^2+ ^(Figure 3F). In marked contrast, EGFP-NEDD4L-C2(+) relocalized to numerous small structures (Figure 3H). Repeating the experiment at a higher data acquisition rate showed that EGFP-NEDD4L-C2(+) transited first from the cytoplasm to the plasma membrane and then to similar small structures (Figure 4A–C). The same mobilization pattern was observed when only the C2 domain of NEDD4L was fused to EGFP (Figure 4D–F). However, the use of various organelle markers did not allow the identification of these structures as lysosomes (Lysotracker^® ^Red), Golgi (BODIPY^® ^TR C_5_-ceramide), mitochondria (Mitotracker^® ^Red), endoplasmic reticulum (ER-Tracker™ Red), early endosomes (Transferrin Texas Red^®^), or autophagosomes (mCherry-LC3) (see Additional file 2). The absence of colocalization with transferrin-Texas Red^® ^suggests that Ca^2+ ^mobilization did not target EGFP-NEDD4L-C2(+) to labeled early endosomal compartments, the organelle in which EGFP-NEDD4L-C2(-) partially localized (see Additional file 2, panel E).

### NEDD4L-C2(+) differentially regulates α- and β-ENaC subunits in response to an increase in intracellular calcium

**Figure 5:**
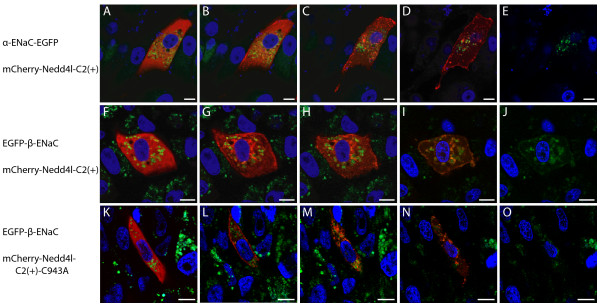
**Distinct subcellular localization behaviors of EGFP tagged α- and β-ENaC and mCherry tagged NEDD4L-C2(+) or NEDD4L-C2(+)-C943A in response to an ionomycin treatment in the presence of Ca^2+^**. Confocal images of live A6 cells stably expressing α-ENaC-EGFP (A-E) or EGFP-β-ENaC (F-J, K-O) transiently transfected with mCherry-NEDD4L-C2(+) (A-E, F-J) or mCherry-NEDD4L-C2(+)-C943A (K-O) and incubated in 1.97 mM Ca^2+^. The images were acquired at 0 (A), 30 (B), 210 (C), 420 (D, E), 0 (F), 41 (G), 66 (H), 236 (I, J), 0 (K), 80 (L), 120 (M), and 240 (N, O)seconds after a 10 μM ionomycin addition. All images are overlays of the blue, green and red channels except for images E, J, and Owhich show only the blue and green channels.

**Figure 6:**
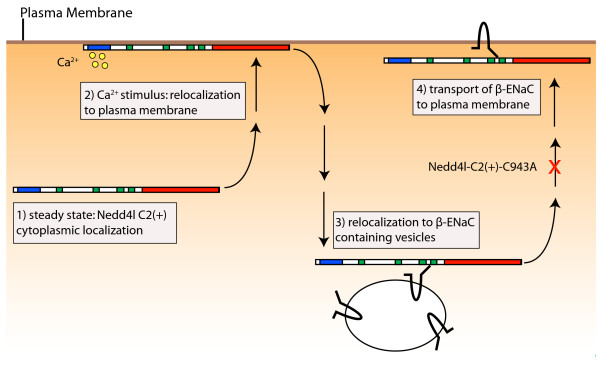
**Summary schematic of the observed mobilization response of NEDD4L-C2(+) in response to a Ca^2+ ^stimulus**. NEDD4L-C2(+)-C943A that lacks ubiquitin ligase activity does not mobilize to the plasma membrane in step 4, and prevents β-ENaC transit to the plasma membrane in step 4.

Observations of the intracellular localization and mobilization of NEDD4L-C2(+) in response to changes in intracellular calcium raised the hypothesis that NEDD4L-C2(+) may affect the intracellular trafficking of its substrate ENaC. This hypothesis was tested by employing two A6 cell lines that stably expressed either the α- or the β-subunit of ENaC fused to EGFP [45]. Because NEDD4L-C2(+) but not NEDD4L-C2(-) relocalized in response to an increase in calcium, the α- and β-ENaC stably-expressing-cell-lines were transiently transfected with NEDD4L-C2(+) that was labeled with the red fluorophore, mCherry (mCherry-NEDD4L-C2(+)). Intracellular trafficking in response to ionomycin and Ca^2+ ^treatment was monitored by time-lapse confocal microscopy. In cells stably expressing α-ENaC-EGFP, mCherry-NEDD4L-C2(+) mobilization occurred in three distinct steps (Figure 5 and see Additional file 3). First, mCherry-NEDD4L-C2(+) moved from the cytoplasm to the plasma membrane (Figure 5A–B). Second, mCherry-NEDD4L-C2(+) transited to the periphery of some of the vesicles containing α-ENaC-EGFP (Figure 5C). Third, mCherry-NEDD4L-C2(+) moved back to the plasma membrane (Figure 5D). Despite partial photo-bleaching of the EGFP fluorophore and fluctuation of the cellular monolayer, the localization of α-ENaC-EGFP remained unaltered throughout the time lapse (Figure 5A–E and see Additional file 3). In cells stably expressing β-ENaC-EGFP, mCherry-NEDD4L-C2(+) mobilization proceeded through the same three distinct steps observed above (Figure 5F–I, Figure 6 and see Additional file 4). A notable and consistent difference however, was that in the third step, β-ENaC relocated to the plasma membrane together with mCherry-NEDD4L-C2(+) (Figure 5I, J and see Additional file 4).

### NEDD4L-C2(+) trafficking of β-ENaC to the plasma membrane requires ubiquitin ligase activity

Mutation of the HECT domain catalytic cysteine residue to alanine abolishes NEDD4L-mediated ubiquitination of α and β-ENaC [47]. To test whether relocation of mCherry-NEDD4L-C2(+) with β-ENaC to the plasma membrane was dependent on mCherry-NEDD4L-C2(+) ubiquitin ligase activity, the cysteine residue (C943) was mutated to alanine to make the construct mCherry-NEDD4L-C2(+)-C943A. Transfection of A6 cells that stably-expressed β-ENaC-EGFP with mCherry-NEDD4L-C2(+)-C943A and time lapse confocal microscopy of live cells following the addition of ionomycin were performed as above (Figure 5K–O and see Additional file 5). In response, mCherry-NEDD4L-C2(+)-C943A first moved from the cytoplasm to the plasma membrane (Figure 5K, L). Then mCherry-NEDD4L-C2(+)-C943A transited to the periphery of some of the vesicles containing β-ENaC-EGFP (Figure 5M). Unlike wild-type mCherry-NEDD4L-C2(+) however, catalytically inactive mCherry-NEDD4L-C2(+)-C943A did not relocate to the plasma membrane (Figure 5N). Also unlike wild-type mCherry-NEDD4L-C2(+) transfected cells, β-ENaC-EGFP did not mobilize to the plasma membrane either (Figure 5O and see Additional file 5).

## Discussion and Conclusion

The availability of two clonal A6 cell lines that stably overexpress functional α- or β-ENaC subunits as EGFP fusion proteins, provided a relevant cellular model to test the effect(s) of potential functional differences between NEDD4L-C2(+) and Neddd4l-C2(-) on the NEDD4L substrates α- or β-ENaC. Furthermore, using the A6 cell culture system allowed functional comparisons in the presence and absence of ENaC subunit overexpression. We report that NEDD4L-C2(+) and NEDD4L-C2(-) exhibit different subcellular localizations and distinct mobilization responses to a calcium stimulus. Additionally, we report that NEDD4L-C2(+) differentially affects short term trafficking of α- and β-ENaC subunits after a calcium stimulus. Moreover, the effect of NEDD4L-C2(+) on a segment of β-ENaC trafficking is ubiquitin dependent.

We observed that the presence or absence of the C2 domain affected the subcellular localization of NEDD4L. NEDD4L-C2(+) and NEDD4L-C2(-) were labeled with EGFP and transfected into A6 cells. Whereas EGFP-NEDD4L-C2(+) localized to the cytoplasm, EGFP-NEDD4L-C2(-) localized to the cytoplasm and early endosome. Although it is unclear what functional significance NEDD4L-C2(-) localization to the early endosome may have, it is plausible that the absence of the C2 domain prevents or hinders NEDD4L-C2(-) targeting to intracellular sites beyond the early endosome.

We observed the mobilization response of EGFP-NEDD4L-C2(+) and EGFP-NEDD4L-C2(-) following an increase in intracellular calcium. A6 cells that had been transfected with either fusion protein were incubated in 1.97 mM Ca^2+ ^and imaged immediately after the addition of 10 μm ionomycin. The Ca^2+ ^concentration is within typical ranges of Ca^2+ ^concentrations that are used in studies that characterize intracellular C2 domain translocation [48,49]. While EGFP-NEDD4L-C2(-) did not respond to the Ca^2+ ^stimulus, EGFP-NEDD4L-C2(+) mobilized from the cytoplasm to the plasma membrane and then to small intracellular compartments. These compartments were not targeted by Transferrin Texas Red^®^, a marker of the early endosome, or other subcellular organelle markers used (see Additional file 2).

The availability of distinct A6 cell lines that stably express α- or β-ENaC, combined with the observation that like NEDD4L-C2(+), ENaC localizes in this overexpression system to unidentified intracellular compartments [45], provided a means and rationale to test the effect(s) of NEDD4L-C2(+) mobilization on a well characterized substrate. Because α- and β-ENaC were stably expressed as EGFP fusion proteins [45], NEDD4L-C2(+) was tagged with the red fluorophore, mCherry. mCherry-NEDD4L-C2(+) exhibited a similar calcium-dependent localization response in cells that expressed either α-ENaC-EGFP or EGFP-β-ENaC. This process occurred in three steps. First, mCherry-NEDD4L-C2(+) moved from the cytoplasm to the plasma membrane. Second, mCherry-NEDD4L-C2(+) transited to the periphery of some of the vesicles that contained ENaC subunits. Third, mCherry-NEDD4L-C2(+) moved back to the plasma membrane. mCherry-NEDD4L-C2(+) did not affect the localization of α-ENaC-EGFP. However, in cells that expressed EGFP-β-ENaC, mCherry-NEDD4L-C2(+) transited to the plasma membrane with EGFP-β-ENaC in the third mobilization step.

Ubiquitination is intimately involved in several aspects of the intracellular trafficking of various proteins [1]. We therefore tested whether the co-transit of NEDD4L-C2(+) and β-ENaC to the plasma membrane in response to an increase in intracellular calcium was ubiquitination dependent. To ablate the ubiquitin ligase capability of NEDD4L-C2(+), the catalytic cysteine 943 (C943) in the HECT domain was mutated to alanine. C943 forms a thioester linkage with ubiquitin prior to substrate ligation and this association is required for substrate ubiquitination. The functional significance of C943 is supported by its conservation in NEDD4L orthologs from humans to yeast. In the context of ENaC regulation, mutation of the catalytic cysteine to alanine abolishes NEDD4L mediated β-ENaC ubiquitination [47]. In the mobilization response to an increase in intracellular calcium, mCherry-NEDD4L-C2(+)-C943A first transited from the cytoplasm to the plasma membrane and then transited to the periphery of some vesicles that contained β-ENaC-EGFP. However, unlike the mobilization response of wild-type mCherry-NEDD4L-C2(+), neither mCherry-NEDD4L-C2(+)-C943A nor β-ENaC-EGFP subsequently transited to the plasma membrane.

The paradigm of E3 enzyme function within the ubiquitin system implies that E3s down-regulate or sequester their substrate targets. Ubiquitination results in substrate transport to the proteasome and lysosome (for degradation) or to endosomal compartments where proteins remain intact but sequestered until needed and recycled for future use [1]. It is not clear how our observation of mCherry-NEDD4L-C2(+) and β-ENaC-EGFP mobilization from intracellular compartments to the plasma membrane would facilitate NEDD4L mediated ENaC down-regulation. Since β-ENaC is active at the cell surface this inside-out movement propels β-ENaC towards its functional locale. This trafficking step may therefore correspond to β-ENaC mobilization from an intracellular pool or recycling. Recent work using live-cell imaging and GFP-labeled ENaC subunits overexpressed in polarized kidney epithelial (MDCK) cells, has shown that cAMP can stimulate rapid ENaC trafficking, more from replenishment than recycling, to the apical surface from an intracellular pool [50]. Furthermore, this rapid cAMP-stimulated replenishment is dependent on the presence of intact PY motifs because it is impaired by PY-motif mutants of ENaC suggesting that the PY motifs play a role in regulating exocytic trafficking of the channel. These results confirmed earlier work in rat thyroid cells demonstrating that cAMP stimulation of ENaC was dependent on the PY motif in the COOH terminus of each subunit, with the most pronounced effect seen with a truncating mutation in the β-ENaC subunit [29].

The observation here that neither catalytically inactive NEDD4L (mCherry-NEDD4L-C2(+)-C943A) nor β-ENaC-EGFP mobilize to the plasma membrane suggests that ubiquitination is necessary for wild-type NEDD4L (mCherry-NEDD4L-C2(+)) and β-ENaC-EGFP co-transit to the plasma membrane. This result is surprising considering that ubiquitination of ENaC as well as myriad other proteins typically leads to internalization and functional down-regulation [10,51]. However, our observation of ubiquitin-dependent mobilization of β-ENaC by NEDD4L-C2(+) provides a novel mechanism for the PY motif-dependent ENaC mobilization from intracellular pools by cAMP. Multiple reports suggest that intracellular calcium oscillations can be mediated by cAMP in renal collecting duct cells [52-54]. A cAMP mediated increase in intracellular calcium could trigger NEDD4L-C2(+) mobilization and targeting of intracellular compartments that contain β-ENaC. Subsequent co-transiting of NEDD4L-C2(+) with β-ENaC from intracellular pools to the cell surface could occur in a PY motif-dependent manner due to the requirement for interactions with NEDD4L-C2(+) WW domains.

The observation that mCherry-NEDD4L-C2(+) mobilizes to the plasma membrane independently in A6 cells that stably express α-ENaC (Figure 5D, see Additional file 3) suggests that fluorescently labeled ENaC subunits may not be the only substrate(s) that are ubiquitinated. Future investigations that employ alternate experimental approaches should provide more detailed insight into the mechanisms of NEDD4L and ENaC trafficking. Likewise, the functional effects of the observed co-mobilization between mCherry-NEDD4L-C2(+) and β-ENaC should be correlated with functional sodium transport studies.

The differential trafficking effect of NEDD4L-C2(+) on α- and β-ENaC subunits is consistent with differential subunit trafficking observed in other contexts. In A6 cells an increase in the density of β-ENaC subunits, but not α- or γ-ENaC subunits, at the cell membrane in response to aldosterone and vasopressin stimuli has been observed [55]. Aldosterone-mediated-regulation of ENaC occurs at least in part by activating the serum glucocorticoid-inducible kinase 1 (Sgk1) [56]. Subsequent phosphorylation of NEDD4L hinders the interaction between NEDD4L and ENaC thereby reducing the negative regulatory effect of NEDD4L on ENaC [57,58]. In vivo vasopressin induces a significant increase of the β-ENaC subunit compared to the α-ENaC subunit in rat kidney [59] as well. Furthermore, a model that describes intracellular ENaC trafficking proposes that its subunits are transported individually, independent of each other, in a non-coordinate manner [60].

The differential regulation of intracellular trafficking as a function of the presence or absence of a C2 domain may extend to other substrates of NEDD4L, including the dopamine transporter (dopamine active transporter, DAT) [61], and to other Nedd4-like E3 ligases, including the NEDD4L paralog Nedd4-1 (Nedd4). Moreover, Nedd4-like proteins have been implicated in the process of viral budding due to the potential interaction between Nedd4-like WW domains and viral late domains that contain proline-rich motifs [62]. Viral pathogens could therefore exploit the differential trafficking behavior and intracellular localization between NEDD4L-C2(+) and NEDD4L-C2(-) to suit aspects of their pathogenesis. Two recent publications indicate that NEDD4L-C2(-) can more potently correct human immunodeficiency virus type 1 (HIV-1) release defects in 293T cells compared to NEDD4L-C2(+) [63,64]. We observed that NEDD4L-C2(-) localizes predominately to transferrin-labeled early endosomal compartments instead of the cytoplasm like NEDD4L-C2(+) (Figure 2). A recent study demonstrated that HIV-1 localizes to transferrin-labeled early endosomal compartments [65]. Perhaps the ability of NEDD4L-C2(-) to correct more readily HIV-1 release compared to NEDD4L-C2(+) is the result of the preferential localization of NEDD4L-C2(-) to early endosomal compartments where HIV-1 is present, thereby facilitating interactions that promote viral egress.

Our data support the hypothesis that the G/A variant impacts ENaC-dependent sodium handling in the ASDN by affecting the relative synthesis of NEDD4L-C2(+) and NEDD4L-C2(-). Individual differences in the regulation of sodium balance mediated by this genotype may contribute modest but definite individual differences in the liability to develop essential hypertension through a sodium dependent mechanism. Our cellular observations suggest a mechanism accounting for recently reported clinical correlations between the genotype of the G/A variant and responses to acute sodium loading as well as changes in blood pressure [40]. Greater synthesis of the NEDD4L-C2(+) isoform relative to the NEDD4L-C2(-) isoform may favor apical recycling or mobilization from intracellular pools, thereby generating a greater apical density of ENaC channels in the ASDN through regulation of the β-ENaC subunit, without concurrent effects on the α-ENaC subunit.

The cellular observations provide a framework for future investigations of the potential relationship between genetic variation in *NEDD4L *affecting relative expression of C2-defined isoforms, subcellular trafficking and regulation of ENaC subunits, and whether and how such genetic variation can contribute to individual susceptibility to develop essential hypertension. It is of particular interest whether our observations relate to ENaC activity. Electrophysiological experiments that would investigate amiloride sensitive sodium current in a similar tissue culture system would be a logical approach to resolve this issue. While we exploited published evidence regarding interaction between ENaC and NEDD4L as well as the availability of a cellular model system [45], the functional implications of C2-containing and C2-lacking NEDD4L isoforms may extend beyond blood pressure control to other NEDD4L mediated processes including viral pathogenesis [62,63] and regulation of dopamine transport [61].

## Methods

### Cloning

*Nedd4l *cDNAs were amplified by PCR from mouse hippocampal cDNA and ligated into the TOPO^®^XL TA cloning vector (Invitrogen; K4700-20). The following primers that were used for amplification are oriented 5' to 3': GCTCCATGGCGACCGGGC and CCTGTAGCGTGATTAATTCCA (NM_001114386.1, NEDD4L that contains the C2 domain (NEDD4L-C2(+)): CCGACAGAAGATCCAACCATGGAG and CCTGTAGCGTGATTAATTCCA (NM_031881.2, NEDD4L that lacks the C2 domain (NEDD4L-C2(-)): ATGACAGAGAAGAGGGGGCGG and TTCTGGAATGGGCACGTTGTA (Protein Kinase C alphaC2 (PKCα-C2)): GCTCCATGGCGACCGGGC and GCCTAAATTGTCCACTTTCTC (NEDD4L C2 domain (C2 only)). Amplified sequences were ligated into the EcoRI site of pEGFP or pmCherry (Clontech; PT3051-5, PT3052-5 & 632524).

### Site Specific Mutagenesis

Alteration of cysteine 943 to alanine in NEDD4L-C2(+) was performed by using the QuikChange^® ^II XL Site-Directed Mutagenesis Kit (Stratagene; 200521). The site directed mutagenesis was performed on the mCherry-NEDD4L-C2(+) plasmid with the following primers: AAACTACCCAGAGCTCATACAGCCTTTAATCGCCTTGATTTACC and GGTAAATCAAGGCGATTAAAGGCTGTATGAGCTCTGGGTAGTTT. The mutation was verified by sequencing.

### Cell Culture and Transfections

A6 cells (including those that stably express either α- or β-ENaC) were cultured as recommended (American Type Culture Collection; reference CCL-102) and as previously described [45]. 24 hours prior to transfection the cells were seeded at a density of 80,000 cells per cm^2 ^in 1.7 cm^2 ^chambers (Nunc Brand; 155382). For transfection, the growth medium was removed, the cells were incubated at 27°C in a solution containing 10 μl Lipofectamine 2000 (Invitrogen; 11668-019) and 1.14 pmol of DNA in a final volume of 300 μl Optimem^® ^I (Gibco; 31985-062). Five hours post transfection, the transfection medium was replaced with growth medium and the cells were incubated for 24 hours at 27°C prior to imaging.

The following cellular organelle markers were utilized in colocalization studies under the following conditions: the dsDNA binding stain Hoechst 33342 (nuclear marker, Invitrogen; H3570: working concentration 2.1 ng/μl) was added 30 minutes prior to imaging. The lysosome marker, Lysotracker^® ^Red (Invitrogen; L-7528: working concentration 83 nM), was added 2 hours prior to imaging and was washed once with growth medium immediately before imaging. The Golgi marker BODIPY^® ^TR C_5_-ceramide (Invitrogen; B34400: working concentration of 5 μM) was added 30 minutes prior to imaging. Cells were washed 3 times with growth medium immediately before imaging. The mitochondrial marker, Mitotracker^® ^Red (Invitrogen; M7512: working concentration 167 nM) was added 45 minutes prior to imaging. The endoplasmic reticulum marker, ER-Tracker™ Red (Invitrogen; E34250: working concentration 1 μM) was added 2 hours prior to imaging. The early endosome marker, Transferrin Texas Red^® ^(Invitrogen; T2875: working concentration 20 ng/μl) was added 1 hour prior to imaging. The autophagosome marker, LC-3, was fused to mCherry (Clontech; 632524) and was co-transfected with EGFP-NEDD4L-C2(+) 24 hours prior to imaging at a quantity of 0.57 pmol each.

In the mobilization experiments, A6 cells that had been transfected as above were incubated in growth medium that contained 1.97 mM Ca^2+^. Ionomycin was added at a working concentration of 10 μm, and the time lapse confocal microscopy was performed immediately.

### Microscopy

Images were acquired using a Fluoview™ FV1000 confocal microscope (Olympus, 60× oil immersion) and the FV10-ASW software. Single plane, xy scans and time lapse, xyt scans were used to image live, transfected A6 cells at 27°C in growth medium. The wavelengths of the excitation lasers were 405 nm and 488 nm for Hoechst 33342 and EGFP respectively. The 543 nm laser was used to excite mCherry as well as the red colocalization fluorophores applying the manufacturers' recommended excitation and emission wavelengths.

### Western Blot

A6 cells were transfected as described, proportionally scaling up all reagents for 3.8 cm^2 ^culture chambers. Cells were isolated by scraping and centrifuging at 14,000 RPM for 5 minutes. The growth medium was then replaced with 80 μl of 1× SDS loading buffer (Laemmli buffer). Following four minutes in a boiling water bath, cells were further homogenized using a Dounce homogenizer. Following a brief centrifugation, 25 μl homogenate was loaded onto a 4–15% Tris-HCl gradient gel (BioRad; 161–1104). Electrophoresis was performed for two hours at 90 V. Proteins were transferred to a nitrocellulose membrane (Amersham; RPN2020E) and incubated with the monoclonal mouse anti-EGFP, JL-8 antibody (Clontech; 632380). A horseradish peroxidase based secondary detection system (Biorad; 170–5043) enabled visualization of bands via chemiluminescence.

## Authors' contributions

NFG performed all experiments and analyses and participated in the design of the study and manuscript preparation. BBY provided the stably transformed A6 cell lines and assistance with manuscript preparation. RBW, JML and AR participated in the design of the study, experimental analyses and preparation of the manuscript. All authors read and approved the manuscript.

## Supplementary Material

Additional file 1 **EGFP-NEDD4L-C2(-) does not colocalize with markers of mitochondria, the endoplasmic reticulum (ER), the Golgi complex or lysosome**. Confocal images of live A6 cells that were transiently transfected with EGFP-NEDD4L-C2(-) (**A-L**) and incubated in the presence of MitoTracker^® ^Red (**A-C**), ER-Tracker™ Red (**D-F**), BODIPY^® ^TR C_5_-ceramide complexed to BSA (Golgi marker) (**G-I**) or Lysotracker^® ^Red (**J-L**). Blue and green channel overlay (**A, D, G, J)**. Blue and red channel overlay (**B, E, H, K)**. Blue, green and red channel overlay (**C, F, I, L**). Scale bars are equivalent to 10 μm. Click here for file

Additional file 2 **In response to a Ca^2+ ^stimulus, EGFP-NEDD4L-C2(+) does not target the endoplasmic reticulum, lysosome, Golgi complex, mitochondria, early endosome or autophagosome**. Confocal images of live A6 cells transiently transfected with EGFP-NEDD4L-C2(+), treated with 10 uM ionomycin and 1.97 mM Ca^2+^, and incubated in the presence of ER-Tracker™ Red (**A**), Lysotracker^® ^Red (**B**), BODIPY^® ^TR C_5_-ceramide complexed to BSA (Golgi marker) (**C**), MitoTracker^® ^Red (**D**), or Transferrin-Texas Red^® ^(**E**). A confocal image of an A6 cell that was transiently cotransfected with EGFP-NEDD4L-C2(+) and mCherry-LC3 (autophagosome marker) after a Ca^2+ ^stimulus (**F**). Blue, green and red channel overlay (**A**-**F**). Scale bars are equivalent to 10 μm. Click here for file

Additional file 3 **Subcellular localization of EGFP tagged α-ENaC and mCherry-NEDD4L-C2(+) in response to ionomycin treatment in the presence of Ca^2+^**. The movie, taken at 1 frame every 30 seconds, is of an A6 cell that stably expresses α-ENaC-EGFP, was transiently transfected with mCherry-NEDD4L-C2(+), and was incubated in 1.97 mM Ca^2+ ^and 10 μM ionomycin. It is the same A6 cell that is shown in Figure 5, panels A-E. Click here for file

Additional file 4 **Subcellular localization of EGFP tagged β-ENaC and mCherry-NEDD4L-C2(+) in response to ionomycin treatment in the presence of Ca^2+^**. The movie, taken at 1 frame every 2 seconds, is of an A6 cell that stably expresses EGFP-β-ENaC, was transiently transfected with mCherry-NEDD4L-C2(+), and was incubated in 1.97 mM Ca^2+ ^and 10 μM ionomycin. It is the same A6 cell that is shown in Figure 5, panels F-J. Click here for file

Additional file 5 **Subcellular localization of EGFP tagged β-ENaC and mCherry-NEDD4L-C2(+)-C943A in response to ionomycin treatment in the presence of Ca^2+^**. The movie, taken at 1 frame every 10 seconds, is of an A6 cell that stably expresses EGFP-β-ENaC, was transiently transfected with mCherry-NEDD4L-C2(+)-C943A, and was incubated in 1.97 mM Ca^2+ ^and 10 μM ionomycin. It is the same A6 cell that is shown in Figure 5, panels K-O. Click here for file
