# Supplementary figures and images for: A human polymorphism affects NEDD4L subcellular targeting by leading to two isoforms that contain or lack a C2 domain
Source: BMC Cell Biol. 2009 Apr 13;10:26. doi: 10.1186/1471-2121-10-26 (PMC2678989; doi:10.1186/1471-2121-10-26)

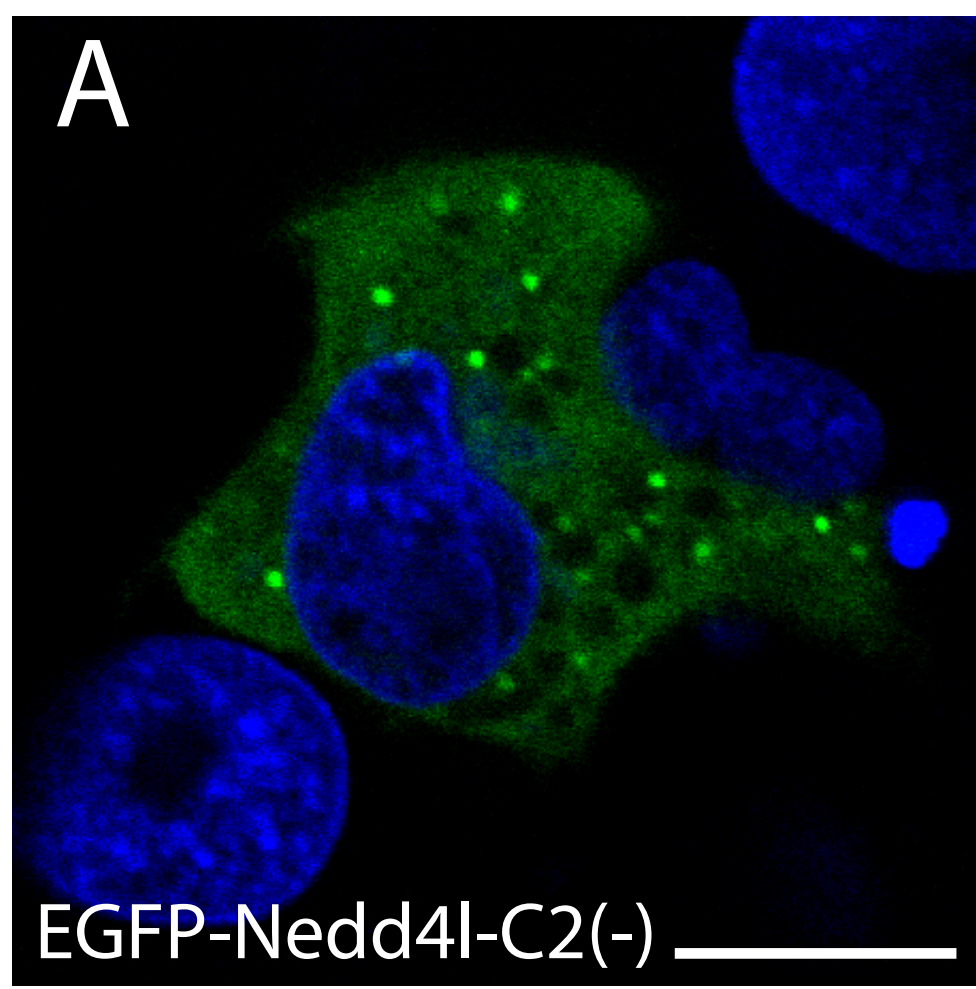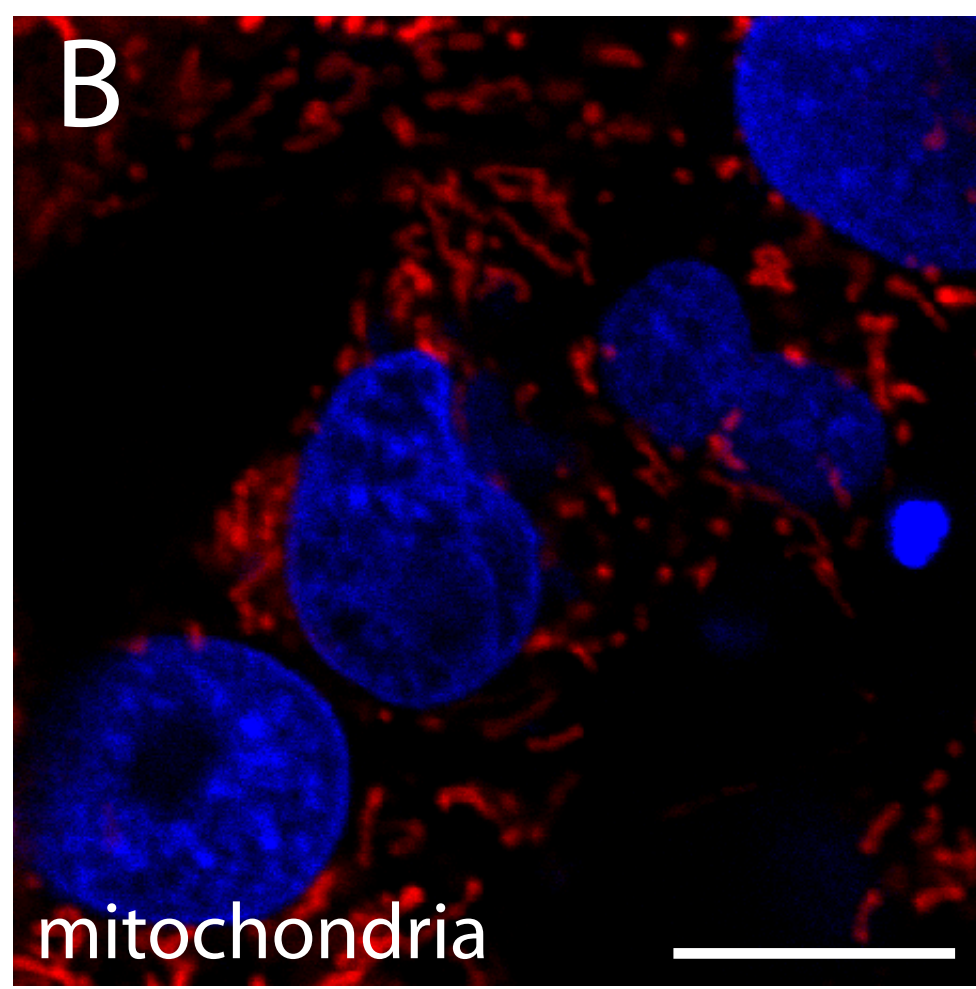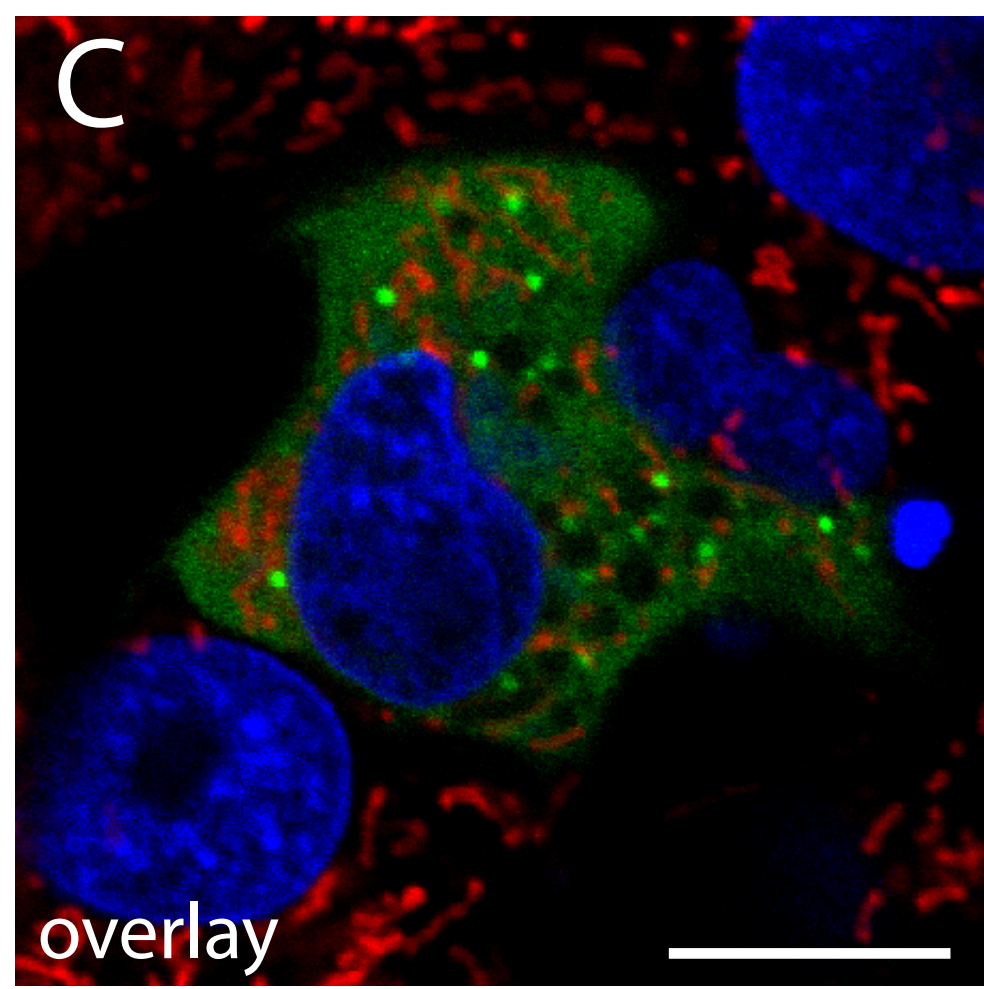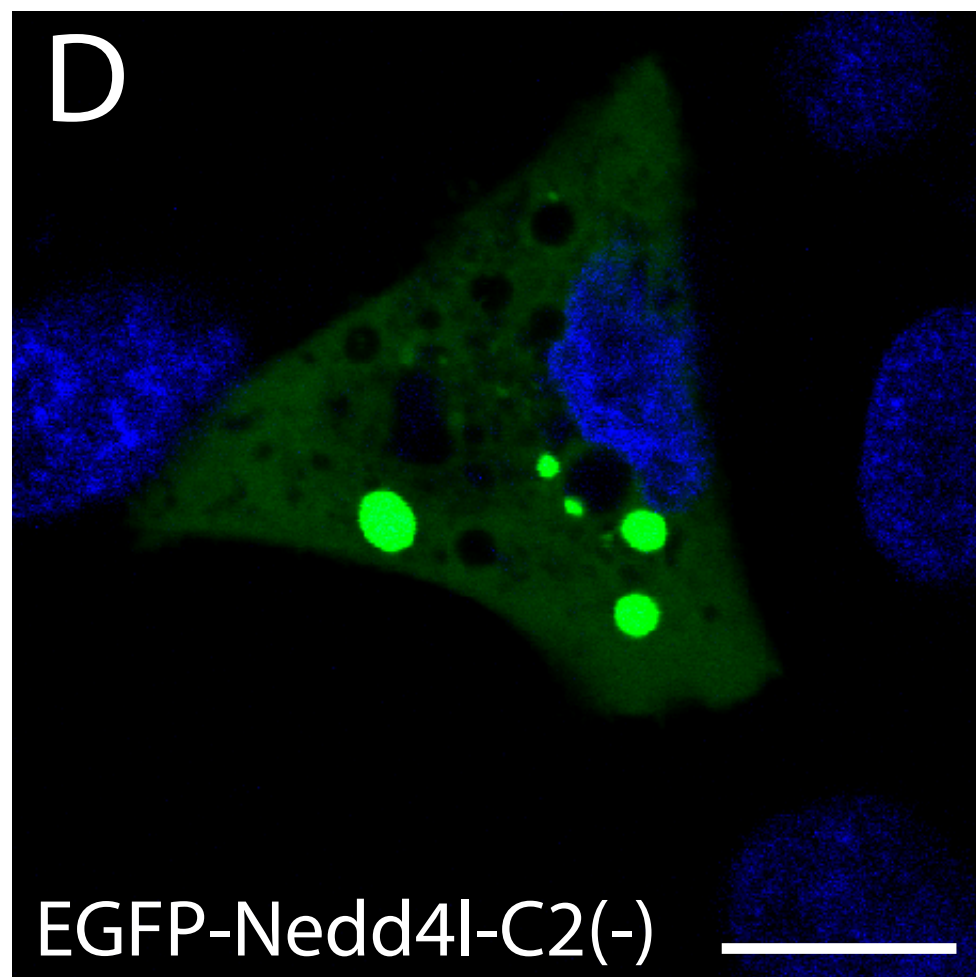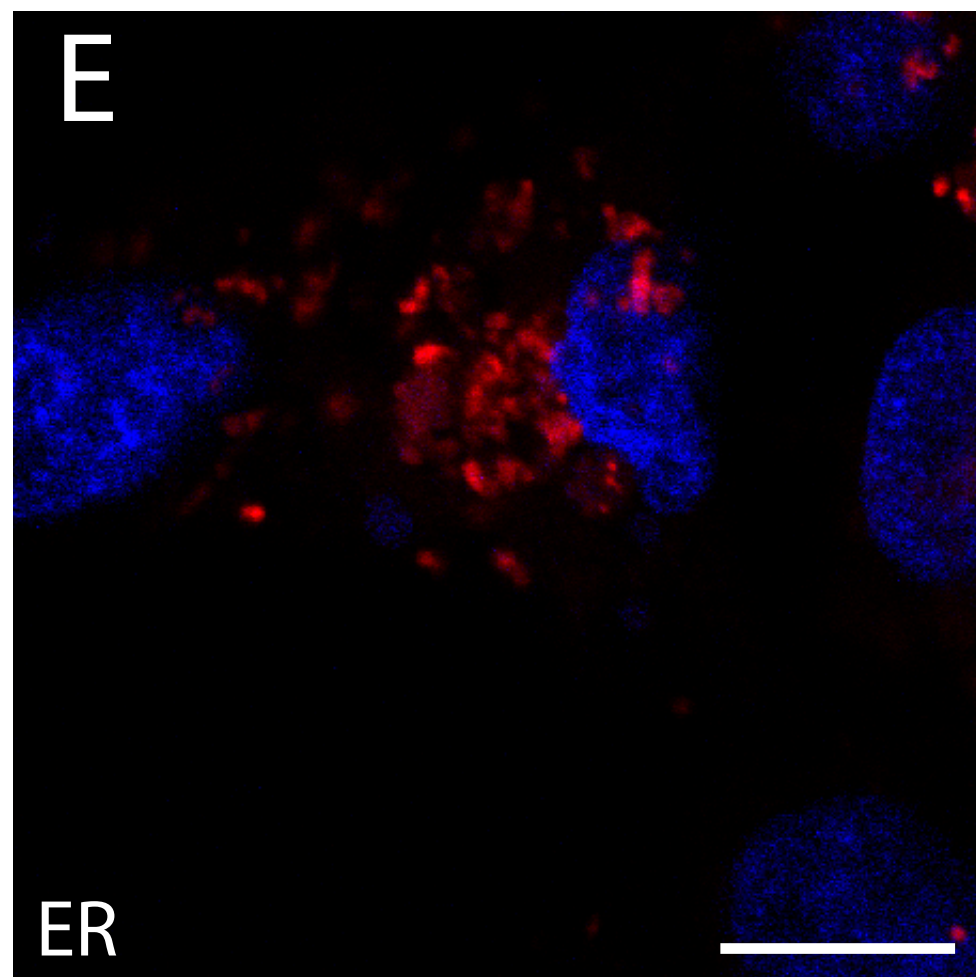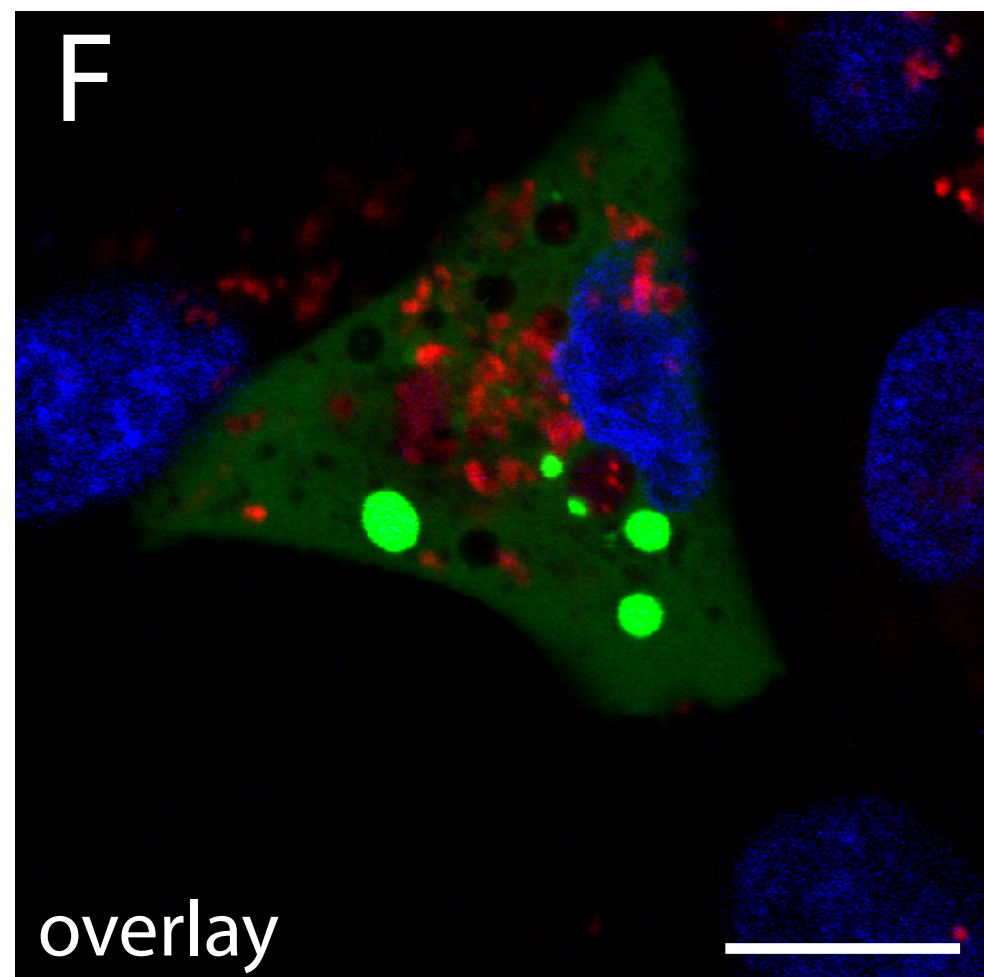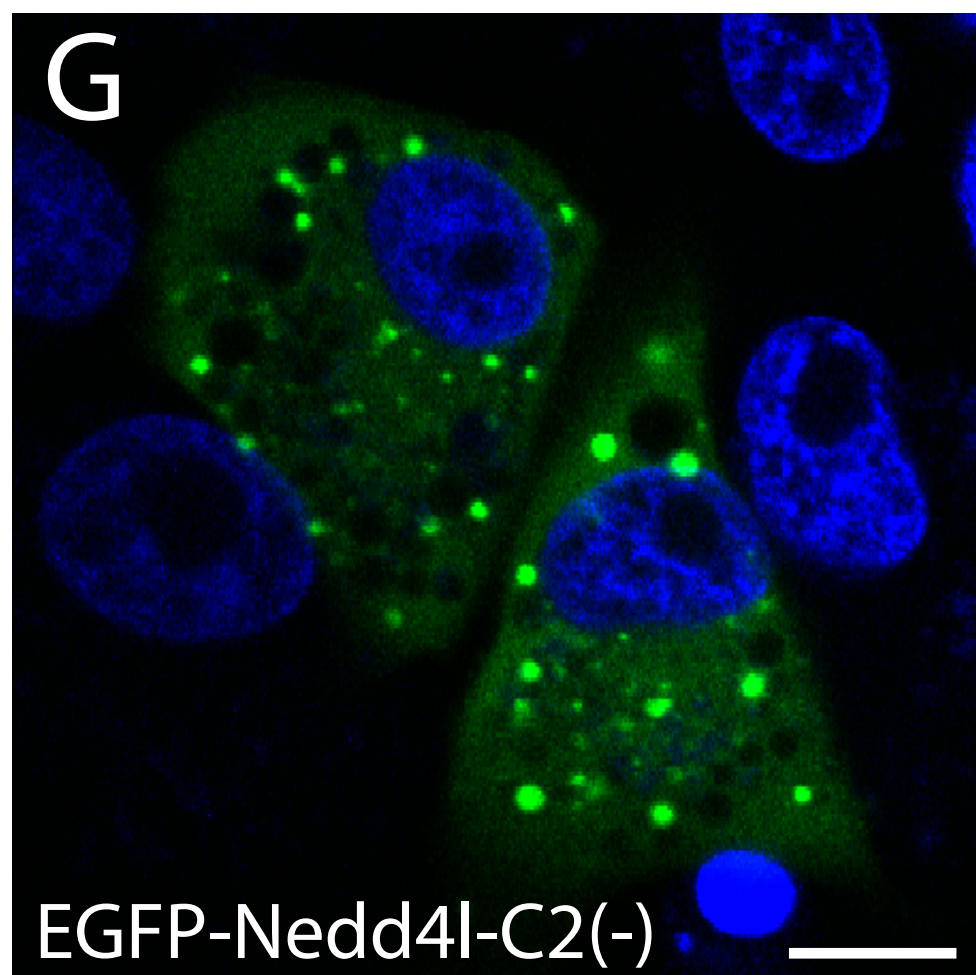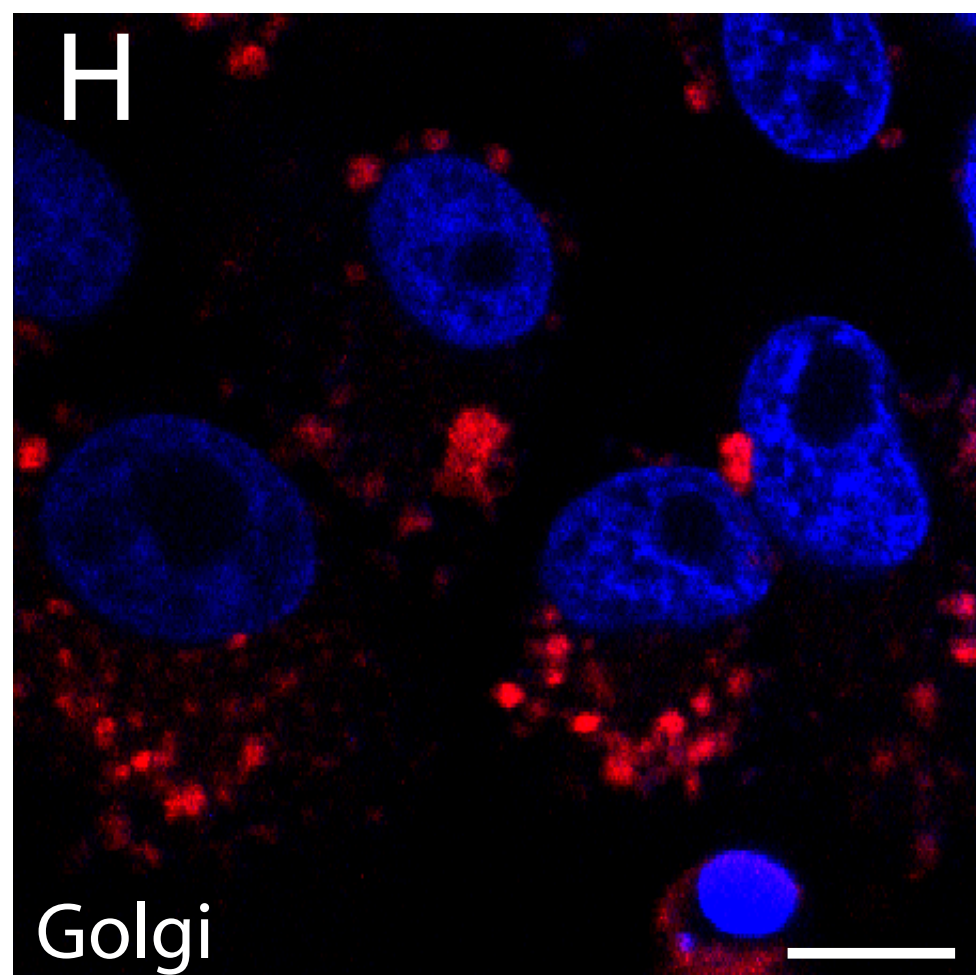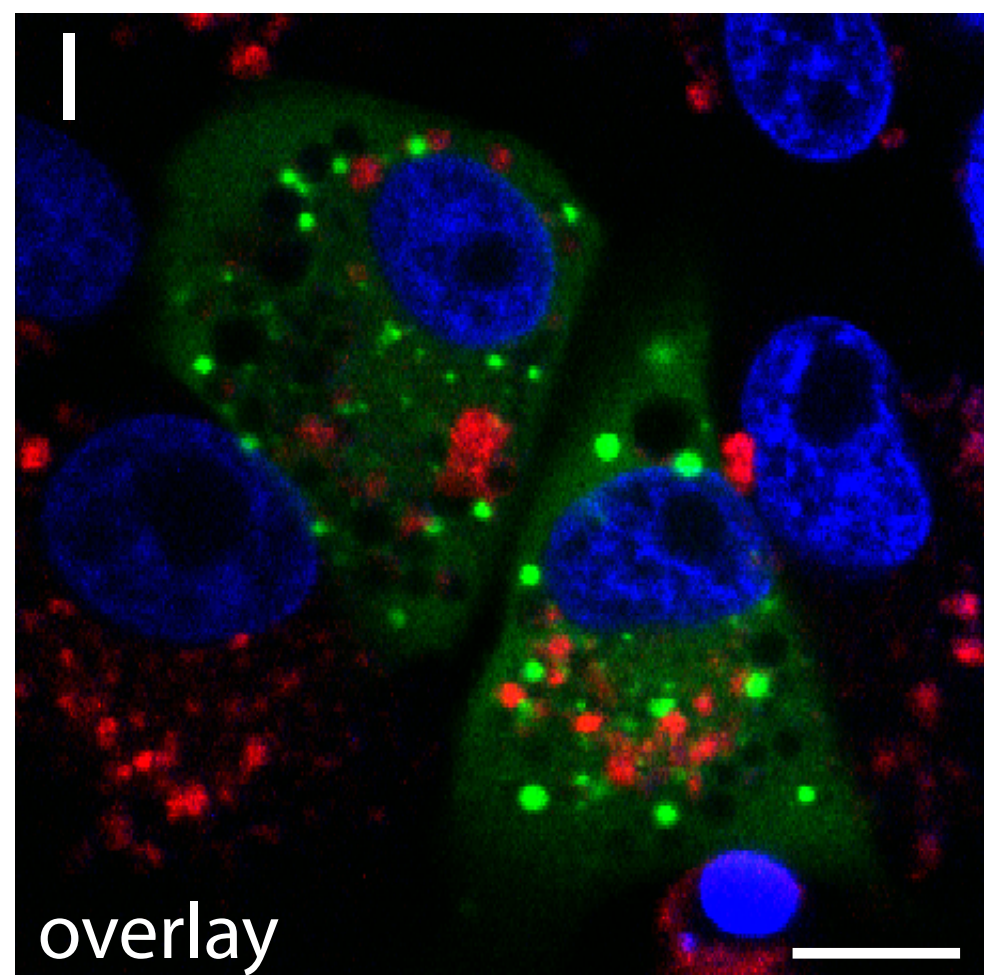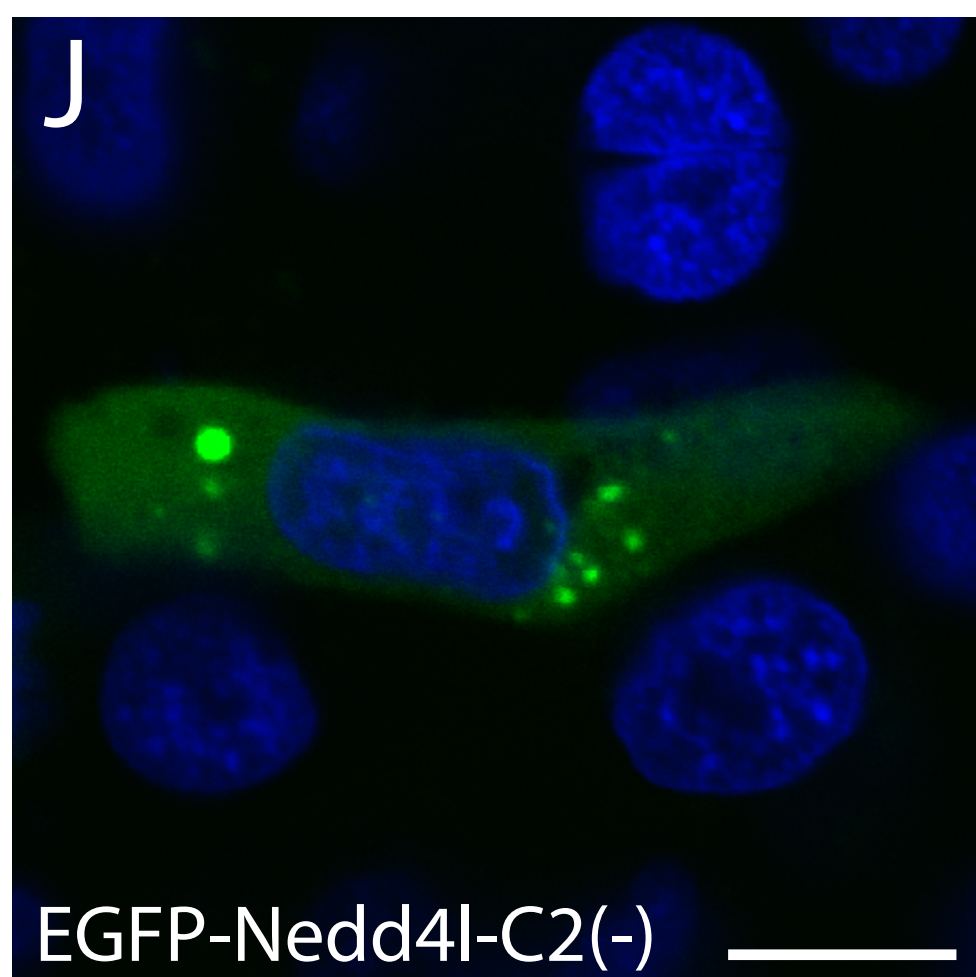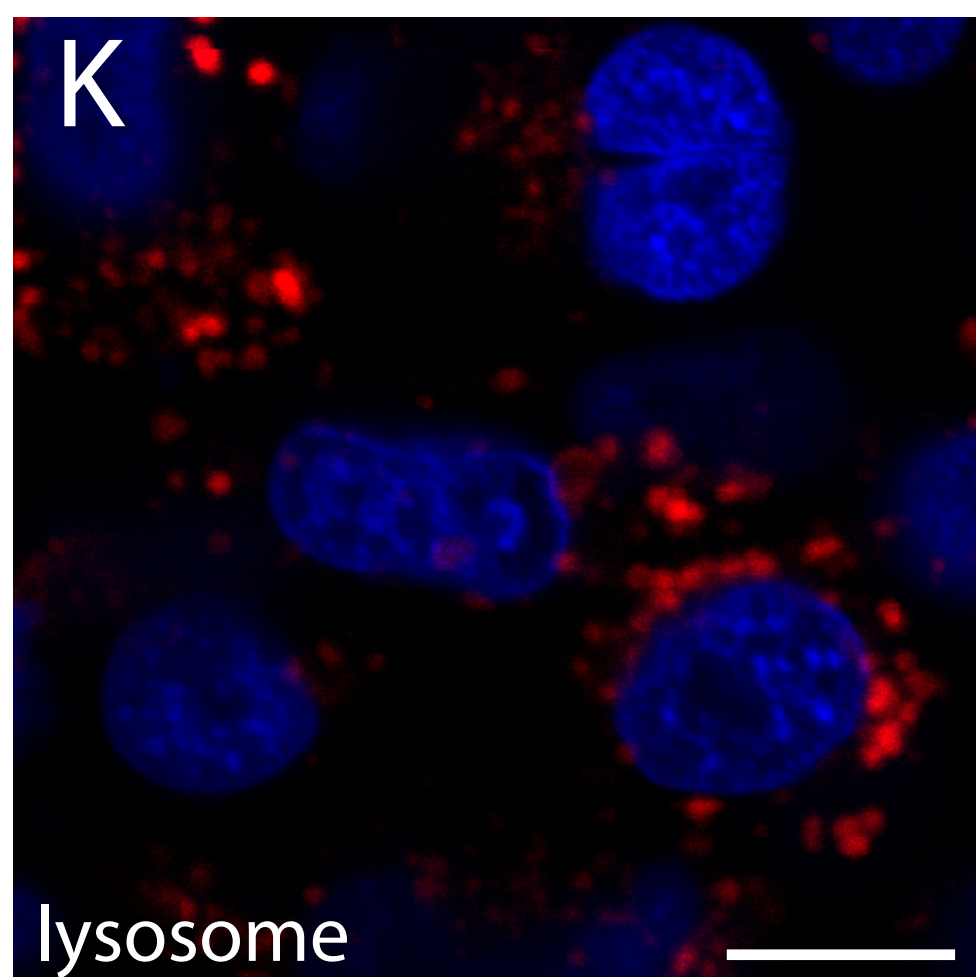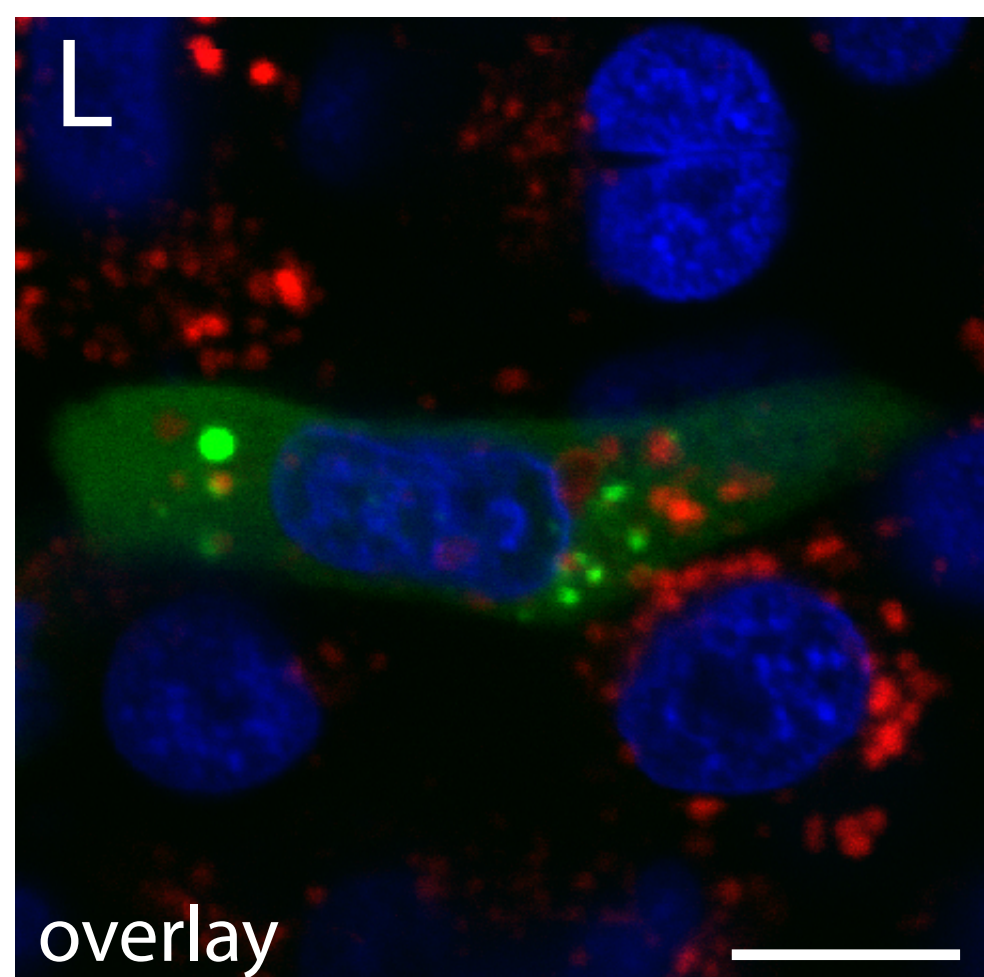

Supplement: Additional file 1 — EGFP-NEDD4L-C2(-) does not colocalize with markers of mitochondria, the endoplasmic reticulum (ER), the Golgi complex or lysosome. Confocal images of live A6 cells that were transiently transfected with EGFP-NEDD4L-C2(-) (A-L) and incubated in the presence of MitoTracker® Red (A-C), ER-Tracker™ Red (D-F), BODIPY® TR C5-ceramide complexed to BSA (Golgi marker) (G-I) or Lysotracker® Red (J-L). Blue and green channel overlay (A, D, G, J). Blue and red channel overlay (B, E, H, K). Blue, green and red channel overlay (C, F, I, L). Scale bars are equivalent to 10 μm. [file 1471-2121-10-26-S1.pdf]

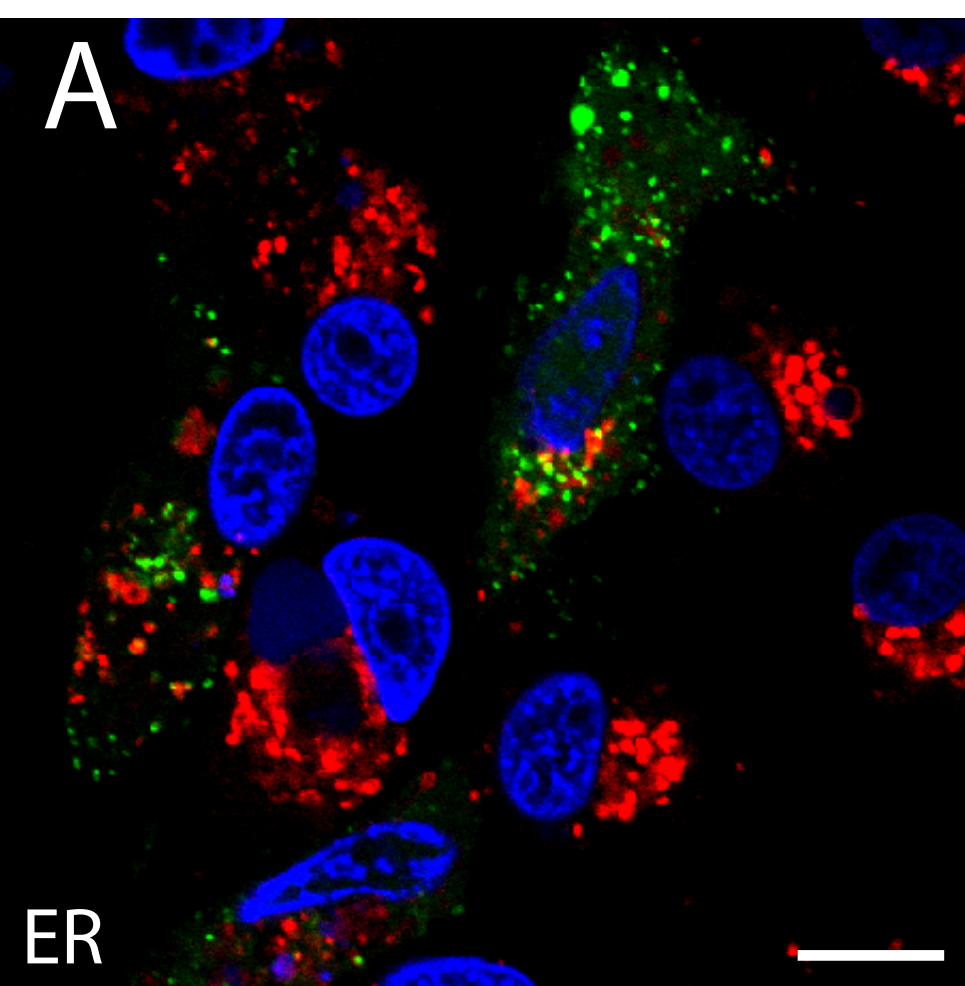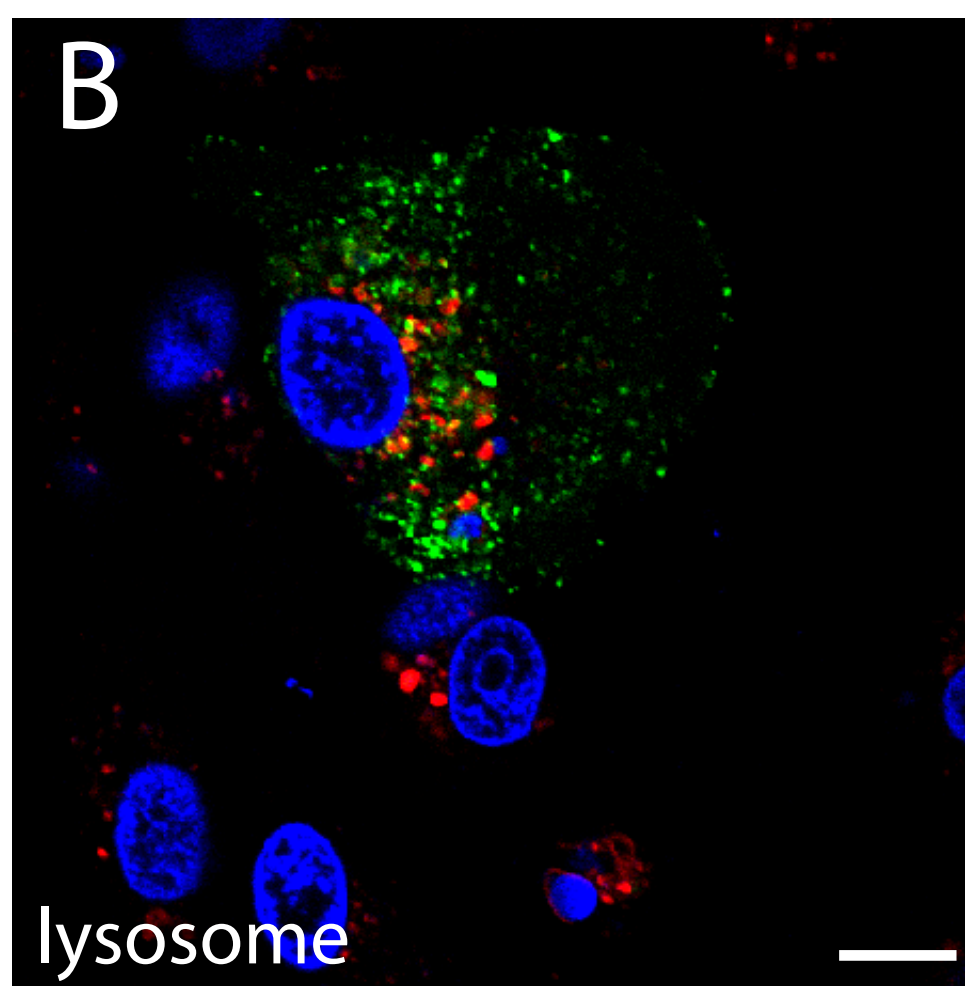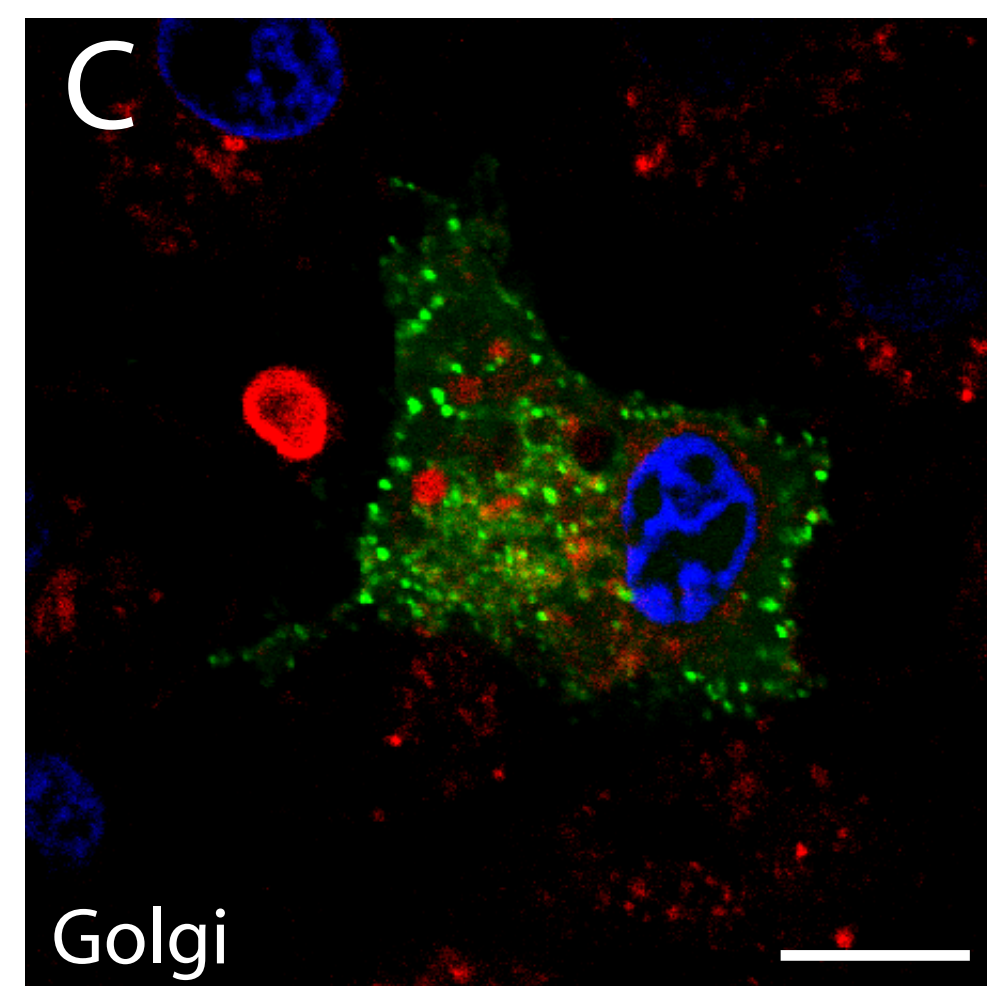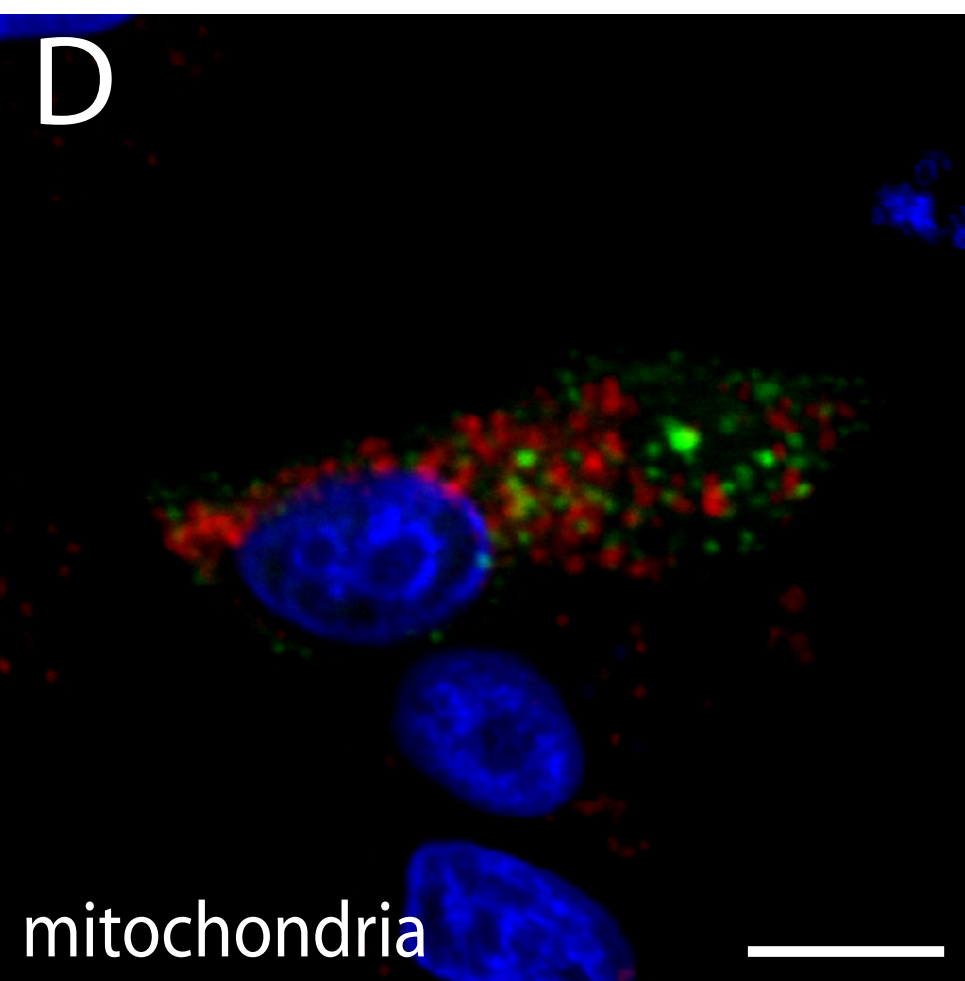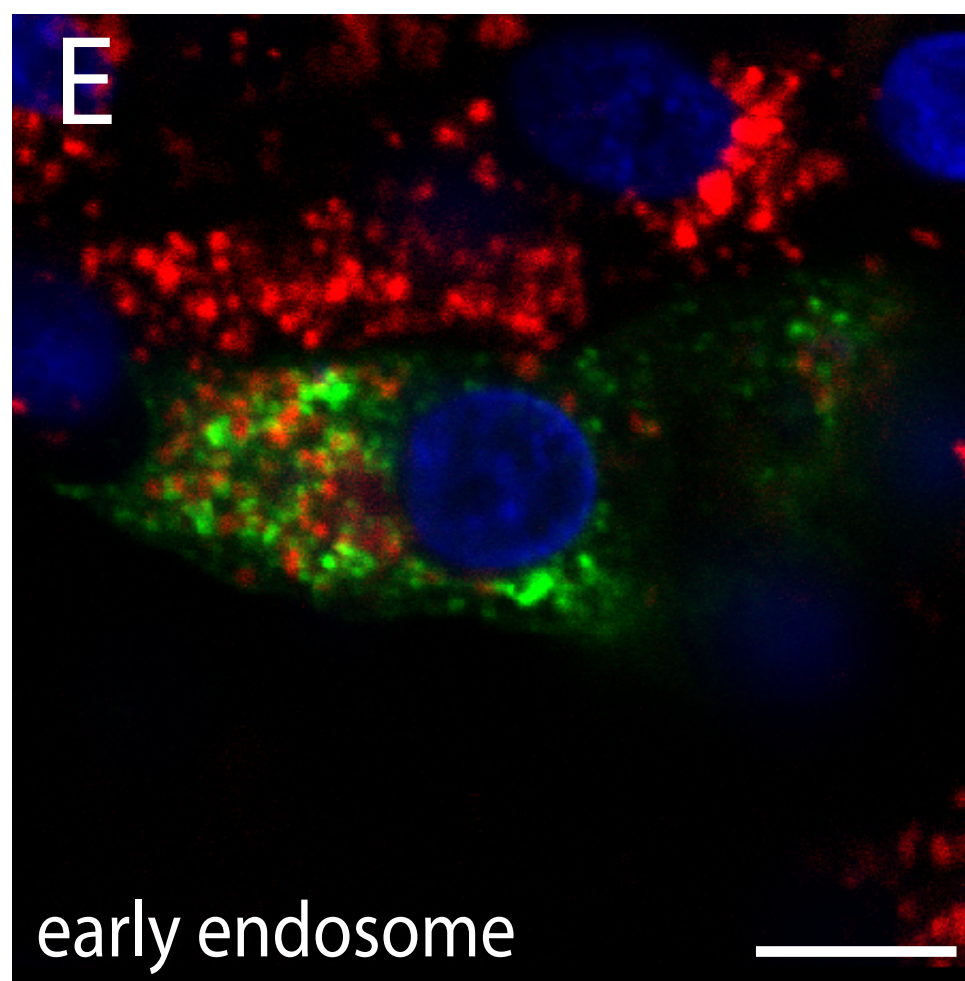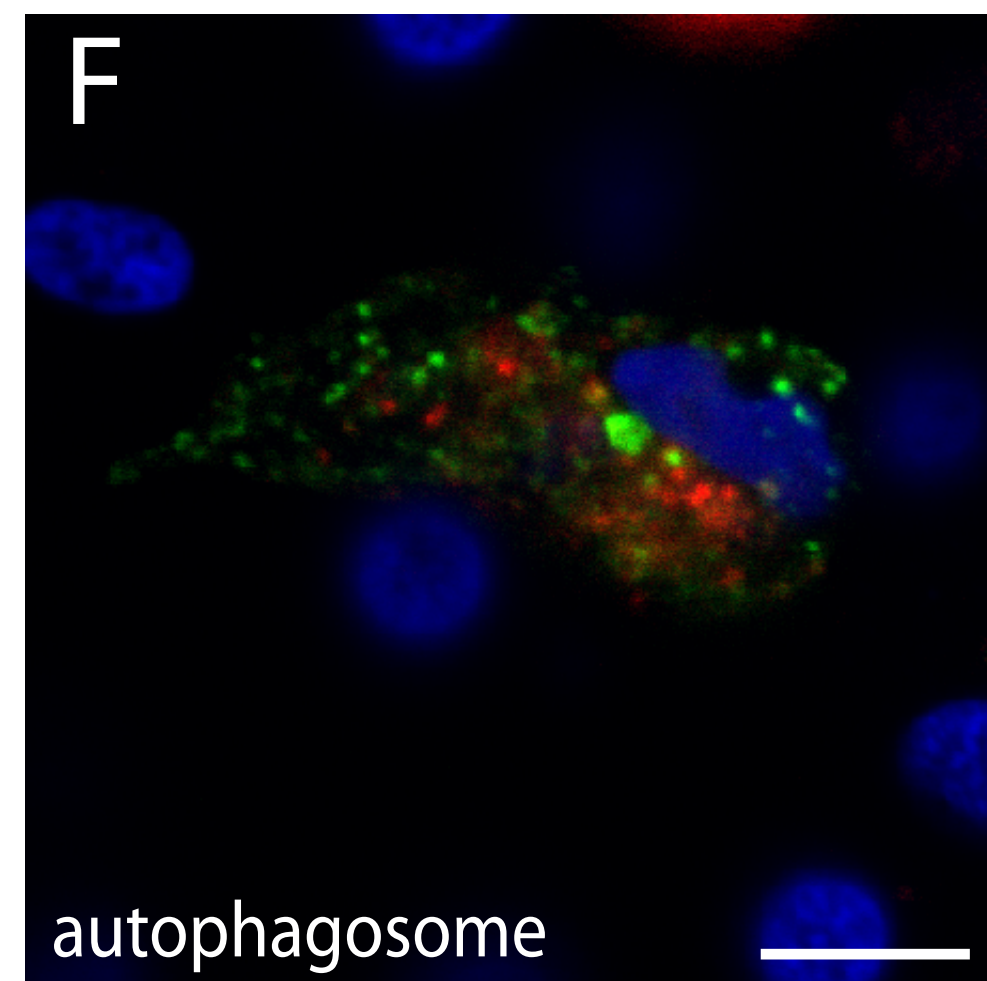

Supplement: Additional file 2 — In response to a Ca2+ stimulus, EGFP-NEDD4L-C2(+) does not target the endoplasmic reticulum, lysosome, Golgi complex, mitochondria, early endosome or autophagosome. Confocal images of live A6 cells transiently transfected with EGFP-NEDD4L-C2(+), treated with 10 uM ionomycin and 1.97 mM Ca2+, and incubated in the presence of ER-Tracker™ Red (A), Lysotracker® Red (B), BODIPY® TR C5-ceramide complexed to BSA (Golgi marker) (C), MitoTracker® Red (D), or Transferrin-Texas Red® (E). A confocal image of an A6 cell that was transiently cotransfected with EGFP-NEDD4L-C2(+) and mCherry-LC3 (autophagosome marker) after a Ca2+ stimulus (F). Blue, green and red channel overlay (A-F). Scale bars are equivalent to 10 μm. [file 1471-2121-10-26-S2.pdf]
